# Acromial Morphology and Subacromial Architecture: A Preliminary Osteological Study

**DOI:** 10.7759/cureus.117674

**Published:** 2026-10-09

**Authors:** Harshika Khatri, Anjulata Rai, Bidyarani D Loitongbam, Jyoti Arora

**Affiliations:** 1 Anatomy, Atal Bihari Vajpayee Institute of Medical Sciences and Dr. Ram Manohar Lohia Hospital, New Delhi, IND

**Keywords:** acromion, bigliani classification, osteophytes, scapula, shoulder impingement syndrome

## Abstract

Background

Acromial morphology may influence the bony configuration of the subacromial region, but osteological evidence quantifying this relationship remains limited. This preliminary study evaluated the association between acromial morphology and subacromial area and described related scapular morphometric parameters in dry human specimens.

Materials and methods

A descriptive cross-sectional osteological study was conducted on 40 dry adult human scapulae, comprising 20 right and 20 left specimens, from the departmental osteology museum. Acromia were classified as Bigliani Type I (flat), Type II (curved), or Type III (hooked). Subacromial area, coracoacromial tilt angle, posterior acromial tilt angle, acromion-supraglenoid distance (DA), and coracoacromial distance were assessed using a digital Vernier caliper and ImageJ (National Institutes of Health, Bethesda, MD, USA). Osteophytes were recorded descriptively. Group differences were evaluated using one-way ANOVA; right-left differences were analyzed using a paired t-test.

Results

Type I acromion was identified in 18 (45.0%) specimens, Type II in 12 (30.0%), and Type III in 10 (25.0%). Osteophytes were present in 24 (60.0%) specimens. The subacromial area showed a progressive reduction from Type I to Type III on both sides; Type III had the smallest mean area (right, 433.50 ± 86.01 mm²; left, 429.86 ± 88.43 mm²). Coracoacromial and posterior acromial tilt also decreased across acromial types, whereas DA did not show a statistically significant difference among types. Because of small subgroup sizes, these inferential findings should be considered exploratory.

Conclusions

Hooked acromial morphology was associated with a smaller osteological subacromial area and lower angular measurements in this specimen series. These findings describe structural variation of the coracoacromial arch but do not establish clinical shoulder impingement or rotator cuff disease. Larger cadaveric and imaging studies with demographic, reliability, and clinical correlation are warranted.

## Introduction

Shoulder pain is a common musculoskeletal complaint, with reported population prevalence ranging from approximately 7% to 34%, and it can substantially impair upper-limb function, work participation, and quality of life [1,2]. Subacromial pain and impingement-related presentations are frequently encountered in clinical practice [3]. Anatomically, the subacromial region is bounded superiorly by the acromion and coracoacromial arch and inferiorly by the humeral head. It contains the rotator cuff tendons, particularly the supraspinatus tendon, together with the subacromial-subdeltoid bursa [4]. Variations in the bony configuration of the coracoacromial region may therefore alter the space available to these structures during shoulder motion and may contribute to subacromial narrowing [5].

Acromial morphology represents an important component of this anatomical configuration. Bigliani et al. classified the acromion as Type I (flat), Type II (curved), and Type III (hooked), and a Type IV (convex) variant has since been described [6]. Type III morphology has frequently been associated with reduced subacromial dimensions and rotator cuff pathology, although findings from clinical and imaging studies have not been entirely consistent [7,8]. In addition to categorical acromial shape, quantitative parameters such as acromial tilt, lateral acromial angle, acromion index, critical shoulder angle, and acromiohumeral distance have been investigated, suggesting that acromial orientation and spatial configuration may be as important as morphology alone [7,9,10].

Despite considerable clinical and radiological interest in acromial morphology, relatively few osteological studies have directly quantified subacromial area alongside complementary scapular morphometric parameters. Evaluating dry human scapulae allows direct assessment of bony architecture without the confounding influence of soft-tissue positioning, although it cannot establish clinical impingement or rotator cuff disease. The primary objective of the present preliminary osteological study was therefore to evaluate the association between Bigliani acromial morphology and subacromial area. Secondary objectives were to compare coracoacromial tilt angle, posterior acromial tilt angle, acromion-supraglenoid distance (DA), and coracoacromial distance across acromial types; assess right-left differences in anatomically paired scapulae; and descriptively characterize acromial osteophytes by anatomical location and morphology.

## Materials and methods

Study design, setting, and duration

This descriptive cross-sectional osteological study was conducted in the Department of Anatomy, Atal Bihari Vajpayee Institute of Medical Sciences and Dr. Ram Manohar Lohia Hospital, New Delhi, India, from July 2024 to September 2024. The study was approved by the Institutional Ethics Committee of Atal Bihari Vajpayee Institute of Medical Sciences and Dr. Ram Manohar Lohia Hospital (approval number: IEC/ABVIMS/RMLH/2023/008). Because the study involved pre-existing dry human skeletal material and no living participants, individual informed consent for participation was not applicable.

Study material, provenance, and sampling

The study included 40 dry adult human scapulae comprising 20 anatomically paired sets, with one right and one left scapula from each individual. The specimens were obtained from the pre-existing osteology museum/teaching collection of the Department of Anatomy, Atal Bihari Vajpayee Institute of Medical Sciences and Dr. Ram Manohar Lohia Hospital, New Delhi. No skeletal material was newly procured specifically for the present study. The museum records did not include the age, sex, handedness, occupation, or clinical history of the individuals represented by the specimens.

All available paired scapulae fulfilling the predefined eligibility criteria during the study period were included. Each specimen was examined for preservation and anatomical integrity before morphometric assessment. Scapulae showing fracture, gross deformity, congenital anomaly, pathological destruction, significant erosion, or damage involving the acromion, coracoid process, glenoid cavity, supraglenoid region, or scapular body that could interfere with the intended measurements were excluded. The use of anatomically paired specimens permitted within-individual comparison of right-left morphometric parameters.

Sample size

No a priori power-based sample-size calculation was performed. All eligible scapulae available in the departmental collection during the study period were included. Accordingly, the study was intended to provide a preliminary, exploratory osteological assessment, and subgroup analyses were interpreted cautiously.

Acromial morphology and osteophytes

Acromial morphology was classified according to the Bigliani system as Type I (flat), Type II (curved), or Type III (hooked), based on the contour of the inferior acromial surface. The recorded dataset contained only Types I-III; no Type IV category was recorded. A single trained investigator performed classification and morphometric assessment. Acromial osteophytes were documented descriptively by anatomical location (anteroinferior surface, lateral surface, or acromial facet) and by morphology (traction-type or claw-type), using the osteological descriptions reported in previous studies [11,12]. No inferential association between osteophyte occurrence and acromial type was claimed in the absence of a complete cross-tabulation and formal statistical test.

Morphometric measurements

Linear measurements were obtained using a digital Vernier caliper (precision 0.01 mm; Mitutoyo Corporation, Kawasaki, Japan). The same investigator repeated each measurement three times under standardized conditions, and the mean of the three readings was used for analysis. The DA was defined as the linear distance between the anteroinferior tip of the acromion and the supraglenoid tubercle. Coracoacromial distance was defined as the shortest distance between the coracoid process and the acromion.

Subacromial area and angular measurements

Standardized digital photographs were obtained with the scapula positioned in a fixed orientation, at a constant camera distance, and with a metric reference scale included in the image. Images were calibrated in ImageJ (National Institutes of Health, Bethesda, MD, USA). The subacromial boundary was traced using the predefined acromial, coracoid, and supraglenoid landmarks shown in Figure 1, and the enclosed area was recorded in mm². Coracoacromial tilt angle (α) and posterior acromial tilt angle (β) were measured from the predefined landmark lines shown in Figure 2. Each digital measurement was repeated three times and averaged.

**Figure 1:**
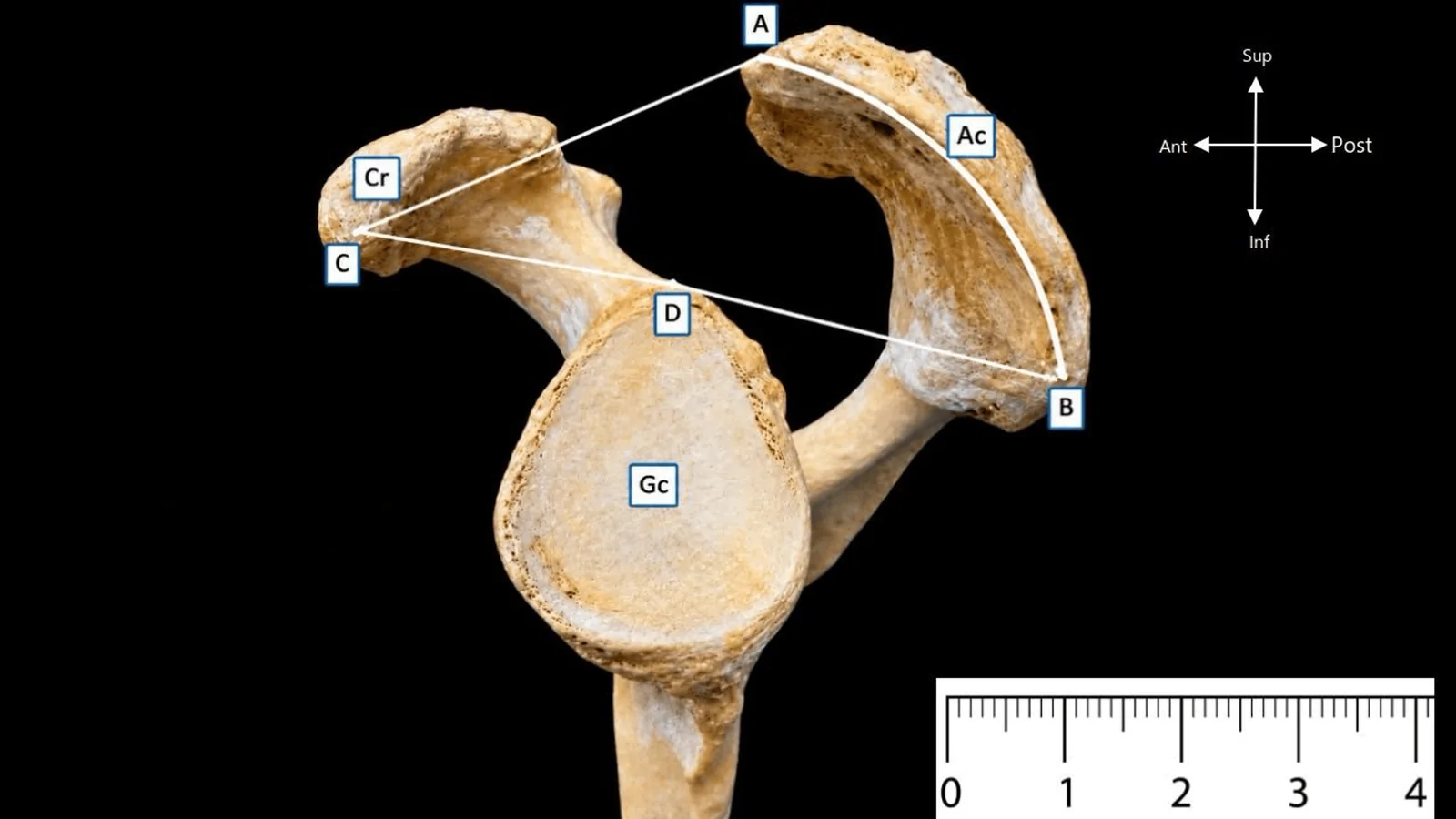
Measurement of the subacromial area on a dry human scapula (left) Lateral view showing the traced boundaries used for ImageJ-based area measurement. A: anterior acromial landmark, B: posterior acromial landmark, C: anterior coracoid landmark, D: supraglenoid reference landmark, Ac: acromion, Cr: coracoid process, Gc: glenoid cavity, S: superior, I: inferior, Ant: anterior, and Post: posterior

**Figure 2:**
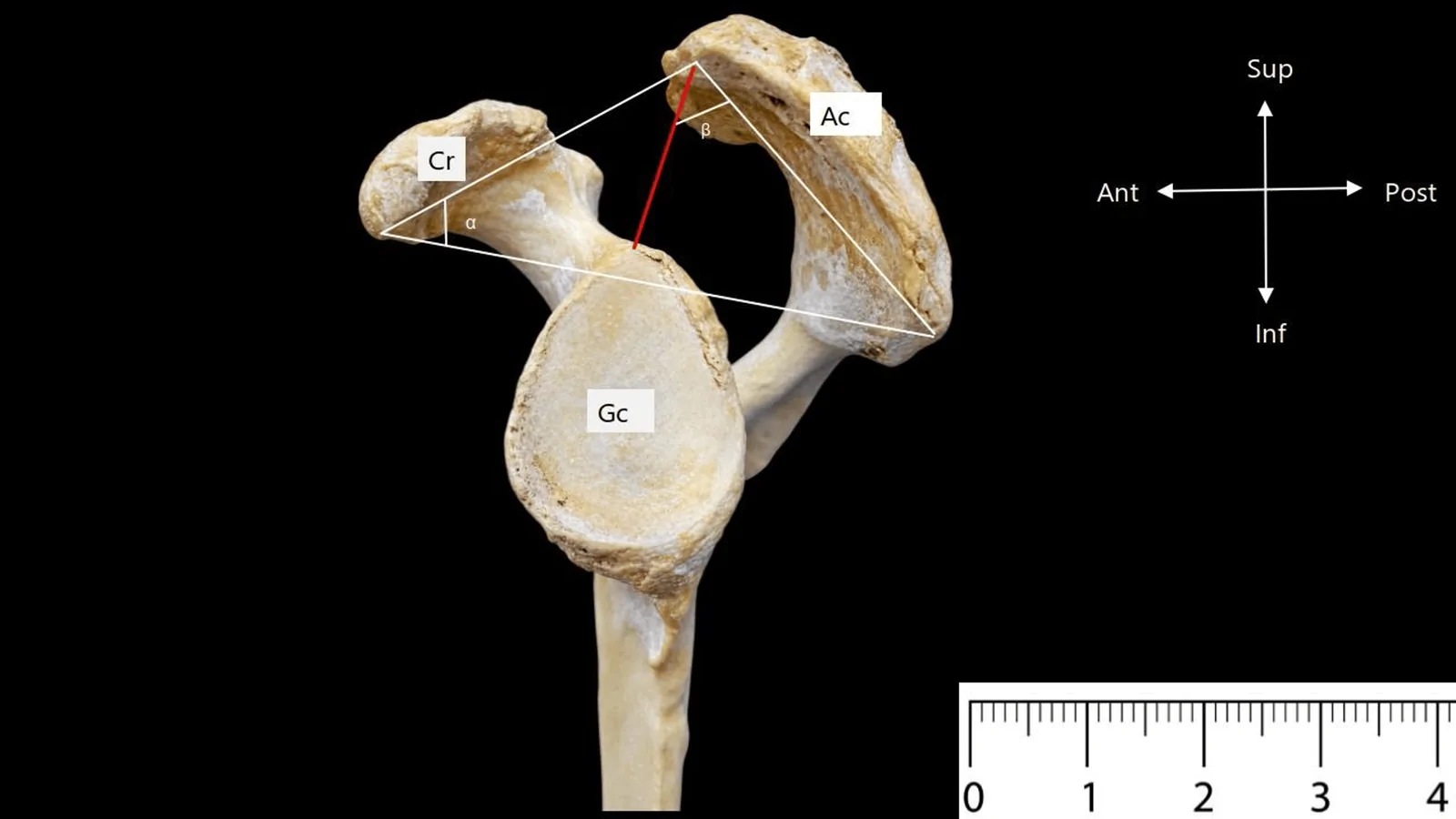
Measurement of coracoacromial tilt angle (α) and posterior acromial tilt (β) The anterior coracoacromial tilt angle (α) is measured by a vertical line that goes across the anterior undersurface of the coracoid and the tip of the supraglenoid tubercle of the glenoid cavity. The posterior acromial tilt (β) is determined by the angle formed by the reference line and a line connecting the most anterior and most posterior points of the acromion's inferior aspects. The red line crosses from the undersurface of the coracoid process to the tip of the supraglenoid tubercle. Co: coracoid, F: acromial facet, Gc: glenoid cavity, Ac: acromion, Ant: anterior, Lat: lateral, Med: medial, Post: posterior, Sup: superior

Quality control

The same investigator repeated all morphometric and ImageJ measurements three times at separate sittings. Standardized positioning, image calibration, and repeated measurements reduced random measurement error. Because one investigator performed all measurements, inter-observer kappa was not applicable. A formal intra-observer intraclass correlation coefficient was not prospectively calculated; this is acknowledged as a limitation.

Statistical analysis

Data were analyzed using SPSS Statistics version 26.0 (IBM Corp. Released 2019. IBM SPSS Statistics for Windows, Version 26.0. Armonk, NY). Continuous variables were summarized as mean ± standard deviation (SD), while categorical variables were presented as frequency and percentage, n (%). Normality of continuous variables within acromial morphology groups was assessed using the Shapiro-Wilk test together with visual inspection of distributions, and homogeneity of variances was evaluated using Levene’s test. Differences in morphometric parameters among Type I, Type II, and Type III acromial groups were primarily analyzed using one-way analysis of variance (ANOVA). For statistically significant omnibus ANOVA results, pairwise comparisons were performed using Tukey’s honestly significant difference post-hoc test when homogeneity of variances was satisfied. When the assumption of equal variances was violated, Welch’s ANOVA with Games-Howell post-hoc comparisons was used. Considering the relatively small numbers within some acromial subgroups, particularly Type III, the corresponding analyses were additionally examined using the Kruskal-Wallis test as a non-parametric sensitivity analysis to assess the robustness of the findings. Effect sizes for omnibus group comparisons were expressed as eta-squared (η²), where applicable. Right-left morphometric measurements obtained from the 20 anatomically paired scapular sets were compared using the paired-samples t-test, with the number of pairs and corresponding degrees of freedom reported. All tests were two-sided, and a p-value <0.05 was considered statistically significant.

## Results

A total of 40 dry human scapulae were examined, including 20 (50.0%) right and 20 (50.0%) left specimens. Type I (flat) acromion was identified in 18 (45.0%) specimens, Type II (curved) in 12 (30.0%), and Type III (hooked) in 10 (25.0%). On the right, the respective distributions were 10 (50.0%), six (30.0%), and four (20.0%); on the left, they were eight (40.0%), six (30.0%), and six (30.0%) (Table 1).

**Table 1:** Distribution of acromial morphology according to the Bigliani classification (N = 40)

Acromion type	Right, n (%) (n = 20)	Left, n (%) (n = 20)	Total, n (%) (N = 40)
Type I (flat)	10 (50.0%)	8 (40.0%)	18 (45.0%)
Type II (curved)	6 (30.0%)	6 (30.0%)	12 (30.0%)
Type III (hooked)	4 (20.0%)	6 (30.0%)	10 (25.0%)
Total	20 (100.0%)	20 (100.0%)	40 (100.0%)

Acromial osteophytes were identified in 24 (60.0%) of 40 specimens. The anteroinferior surface was the most common location for osteophyte formation, followed by the acromial facet and lateral surface (Figures 3-4). Based on morphology, traction-type osteophytes were more frequently encountered than claw-type osteophytes. Osteophytes were more common in scapulae with greater acromial curvature, particularly those with Type III acromial morphology.

**Figure 3:**
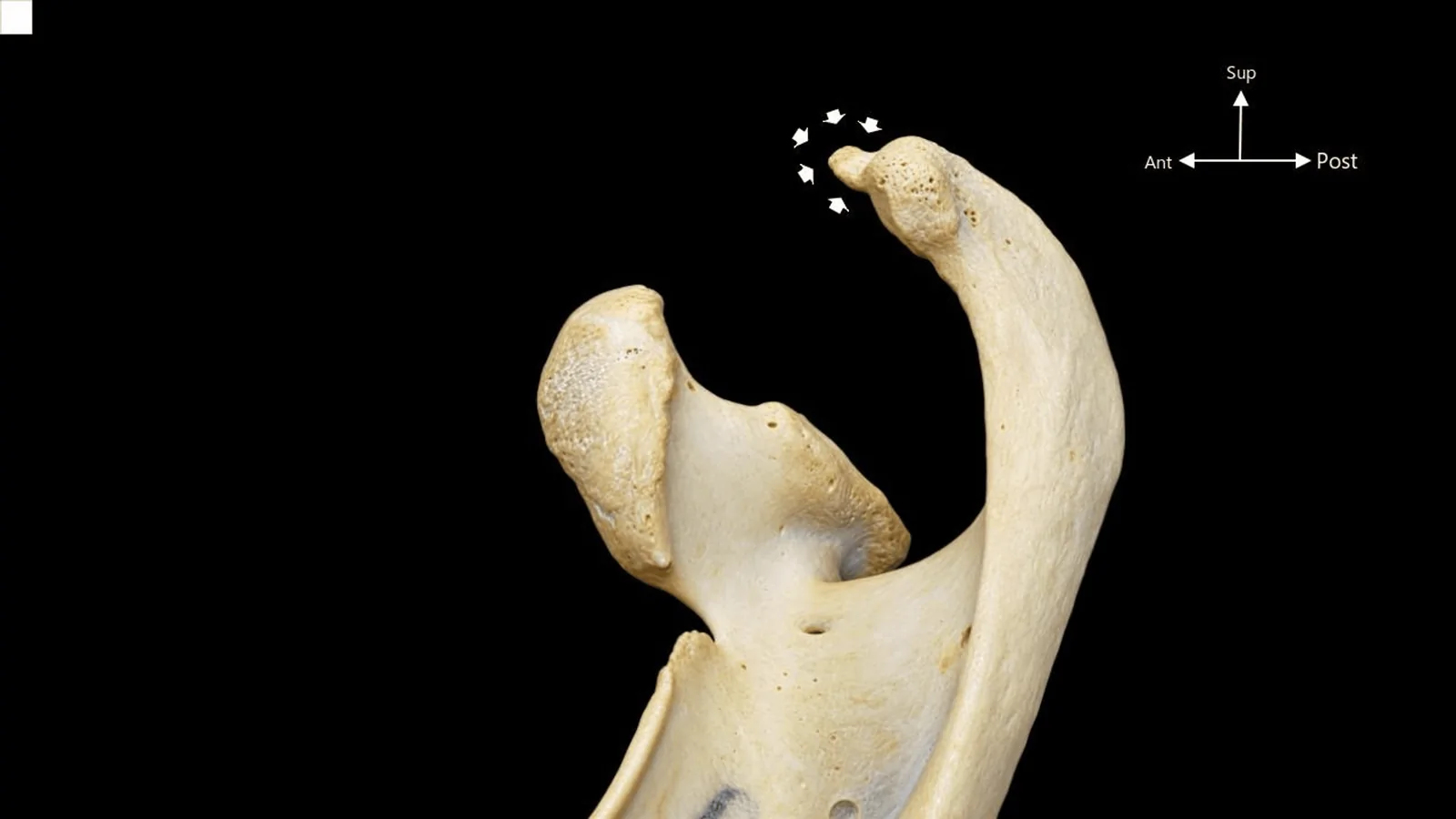
Superior view of a right dry scapula demonstrating an osteophyte (arrowheads) at the acromial facet Ant: anterior, Pos: posterior, Sup: superior

**Figure 4:**
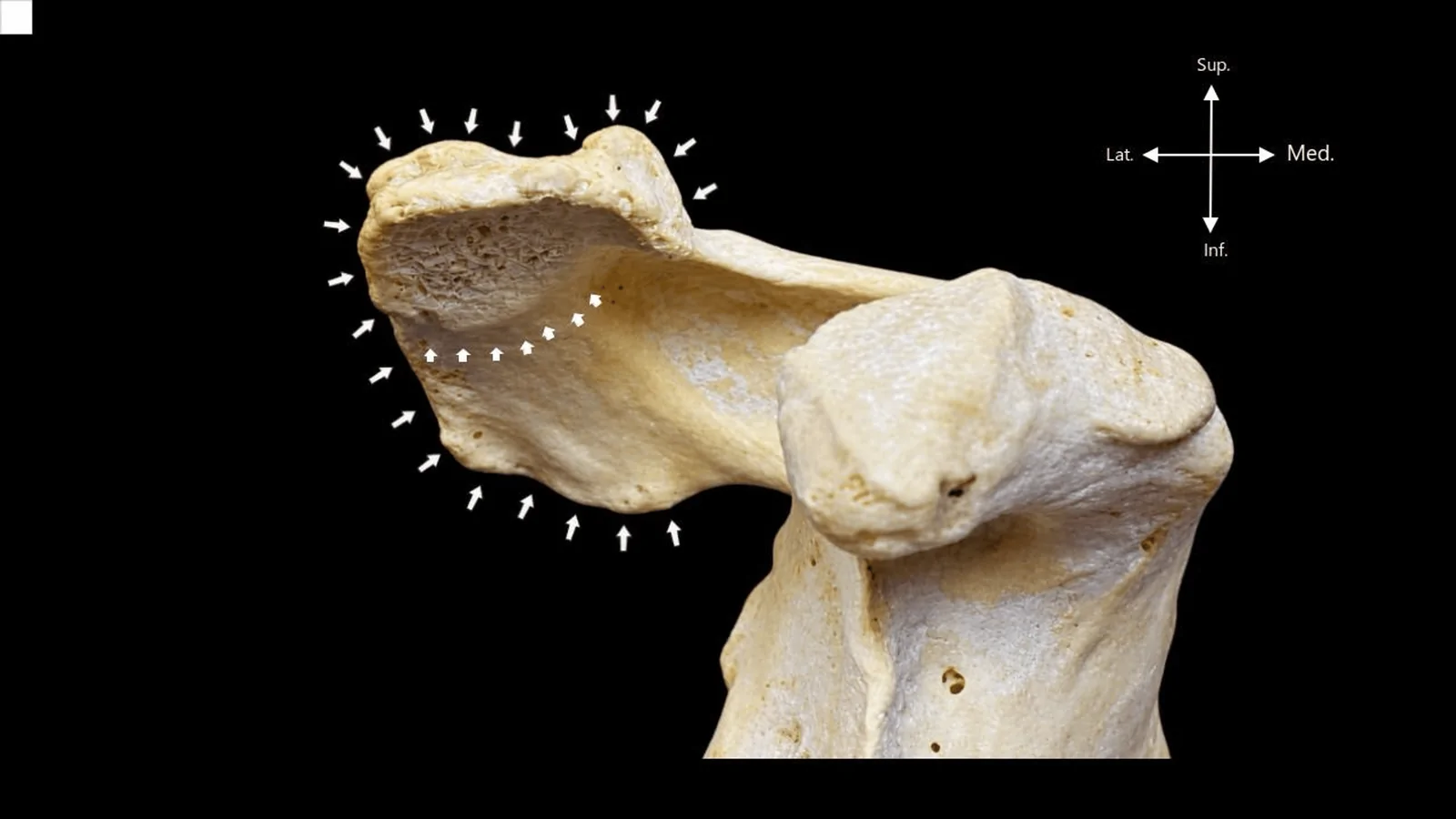
Anterior view of a right dry scapula demonstrating osteophyte formation along the anteroinferior/lateral acromial margin (outlined by arrowheads) Lat: lateral, Med: medial, Sup: superior, Inf: inferior

Table 2 summarizes the side-wise morphometric parameters of the 20 anatomically paired scapular sets. Paired-samples t-tests were used to compare right-left measurements, with 20 paired observations and 19 degrees of freedom for each comparison. No statistically significant side-wise differences were observed for coracoacromial tilt, DA, subacromial area, or coracoacromial distance (all p > 0.05). Posterior acromial tilt was significantly greater on the right than on the left, with a mean paired difference of 1.57° (95% CI: 0.90 to 2.24°; t = 4.90, df = 19; p = 0.0001).

**Table 2:** Comparison of morphometric parameters between anatomically paired right and left scapulae (20 pairs) Values are expressed as mean ± SD. The mean paired difference was calculated as right minus left. Comparisons were performed using the paired-samples t-test with 20 anatomically paired scapular sets (df = 19). † Statistically significant at p < 0.05, CI: confidence interval, DA: acromion-supraglenoid distance, SD: standard deviation

Parameter	Right, mean ± SD	Left, mean ± SD	Mean paired difference (right-left)	95% CI of mean difference	t-value (df = 19)	p-value
Coracoacromial tilt (°)	40.86 ± 5.73	40.92 ± 6.15	−0.06	−0.16 to 0.04	−1.30	0.21
Acromion–supraglenoid distance, DA (mm)	27.07 ± 2.66	26.62 ± 2.36	0.45	−0.26 to 1.16	1.33	0.2
Subacromial area (mm²)	604.72 ± 91.25	608.88 ± 96.79	−4.16	−8.87 to 0.55	−1.85	0.08
Posterior acromial tilt (°)	67.56 ± 5.12	65.99 ± 5.43	1.57	0.90 to 2.24	4.9	0.0001†
Coracoacromial distance (mm)	31.78 ± 5.23	32.70 ± 4.80	−0.92	−4.53 to 2.69	−0.53	0.6

Table 3 summarizes the comparison of subacromial architectural parameters across the three acromial morphology groups. Across acromial types, the subacromial area showed a progressive reduction from Type I to Type III on both sides. On the right, the mean subacromial area decreased from 607.88 ± 48.12 mm² in Type I to 515.75 ± 61.40 mm² in Type II and 433.50 ± 86.01 mm² in Type III acromia. The corresponding left-sided values were 686.50 ± 35.70 mm², 591.48 ± 61.36 mm², and 429.86 ± 88.43 mm², respectively. The overall differences were statistically significant on both the right (F(2,17) = 12.97, p < 0.001, η² = 0.60) and left (F(2,17) = 28.83, p < 0.001, η² = 0.77) sides. Tukey-adjusted pairwise comparisons showed significantly larger subacromial area in Type I than Type II and Type III acromia on both sides; on the left, Type II was also significantly greater than Type III. Coracoacromial tilt showed a similar progressive reduction from Type I to Type III, with significant overall differences on the right (F(2,17) = 25.81, p < 0.001, η² = 0.75) and left (F(2,17) = 33.61, p < 0.001, η² = 0.80). Tukey post-hoc analysis demonstrated significant differences between Type I and both Type II and Type III, whereas the Type II-Type III difference was not statistically significant after adjustment. Posterior acromial tilt also differed significantly among acromial types on the right (F(2,17) = 50.75, p < 0.001, η² = 0.86) and left (F(2,17) = 44.20, p < 0.001, η² = 0.84), with Type I showing significantly greater values than Types II and III. In contrast, DA did not differ significantly among acromial types on either the right (F(2,17) = 1.83, p = 0.191, η² = 0.18) or left (F(2,17) = 1.28, p = 0.305, η² = 0.13) side. Given the relatively small Type III subgroups, particularly on the right, these between-group findings were interpreted as exploratory. Assumption testing and non-parametric sensitivity analyses should be used to confirm the robustness of the observed associations.

**Table 3:** Comparison of subacromial architectural parameters across acromial morphology groups Values are presented as mean ± SD. One-way ANOVA was used for comparison among Type I, Type II, and Type III acromial groups. Significant omnibus ANOVA results were followed by Tukey's honestly significant difference post hoc test. A two-sided p < 0.05 was considered statistically significant. η²: eta-squared effect size, DA: acromion-supraglenoid distance, SD: standard deviation

Parameter	Type I, mean ± SD	Type II, mean ± SD	Type III, mean ± SD	Omnibus ANOVA	Tukey-adjusted post-hoc comparison	η²
Coracoacromial tilt (°), right	45.40 ± 5.38	32.55 ± 2.00	30.93 ± 2.68	F(2,17) = 25.81, p < 0.001	I > II, p < 0.001; I > III, p < 0.001; II vs III, p = 0.824	0.75
Coracoacromial tilt (°), left	46.39 ± 4.57	36.15 ± 2.22	31.17 ± 2.99	F(2,17) = 33.61, p < 0.001	I > II, p < 0.001; I > III, p < 0.001; II vs III, p = 0.066	0.8
DA (mm), right	24.90 ± 3.45	22.23 ± 1.62	23.81 ± 1.17	F(2,17) = 1.83, p = 0.191	Post-hoc testing not indicated	0.18
DA (mm), left	24.08 ± 2.45	23.72 ± 3.11	22.08 ± 1.26	F(2,17) = 1.28, p = 0.305	Post-hoc testing not indicated	0.13
Subacromial area (mm²), right	607.88 ± 48.12	515.75 ± 61.40	433.50 ± 86.01	F(2,17) = 12.97, p < 0.001	I > II, p = 0.023; I > III, p < 0.001; II vs III, p = 0.117	0.6
Subacromial area (mm²), left	686.50 ± 35.70	591.48 ± 61.36	429.86 ± 88.43	F(2,17) = 28.83, p < 0.001	I > II, p = 0.031; I > III, p < 0.001; II > III, p < 0.001	0.77
Posterior acromial tilt (°), right	65.22 ± 1.41	56.10 ± 3.06	52.40 ± 3.41	F(2,17) = 50.75, p < 0.001	I > II, p < 0.001; I > III, p < 0.001; II vs III, p = 0.073	0.86
Posterior acromial tilt (°), left	64.14 ± 2.44	54.55 ± 3.27	50.80 ± 2.62	F(2,17) = 44.20, p < 0.001	I > II, p < 0.001; I > III, p < 0.001; II vs III, p = 0.075	0.84

## Discussion

This preliminary osteological study found that Type I acromion was the most prevalent morphology, occurring in 18 (45.0%) specimens, followed by Type II in 12 (30.0%) and Type III in 10 (25.0%). This distribution is broadly consistent with previous anatomical studies by Singroha et al., Pardeep et al., and Ravindranath et al., which reported flat and curved acromia as common morphological variants. In contrast, hooked Type III acromion was comparatively less frequent [11-13]. Although Type III morphology was less prevalent in the present series, its anatomical configuration is clinically relevant because inferior hooking may reduce the available subacromial region [14]. Nevertheless, because the present investigation involved dry scapulae rather than symptomatic patients, these findings indicate an anatomical predisposition rather than a direct diagnosis or risk estimate for shoulder impingement.

A major finding was the progressive reduction in subacromial area from Type I to Type III morphology. On the right, the mean subacromial area decreased from 607.88 ± 48.12 mm² in Type I to 515.75 ± 61.40 mm² in Type II and 433.50 ± 86.01 mm² in Type III acromia; corresponding left-sided values were 686.50 ± 35.70, 591.48 ± 61.36, and 429.86 ± 88.43 mm². These differences were significant on both sides (right: F(2,17) = 12.97, p < 0.001, η² = 0.60; left: F(2,17) = 28.83, p < 0.001, η² = 0.77), indicating large effect sizes. Tukey-adjusted comparisons demonstrated significantly greater subacromial area in Type I than in Type II and Type III acromia on both sides. The Type II-Type III difference was also significant on the left. These findings support Aragão et al.'s observation that increasing acromial curvature is associated with a reduction in the subacromial region [15]. Anatomically, the inferior hook of a Type III acromion may encroach upon the coracoacromial arch and reduce the space available for the supraspinatus tendon and subacromial-subdeltoid bursa. This pattern is compatible with the extrinsic compression hypothesis and with clinical and radiological studies linking hooked morphology with rotator cuff pathology [16,17]. However, studies by Altuntas et al. and Kaur et al. indicate that rotator cuff disease cannot be attributed to acromial morphology alone and may also reflect age-related degeneration, tendon characteristics, and shoulder biomechanics [8,18].

Coracoacromial tilt and posterior acromial tilt demonstrated similarly marked differences among morphological types. Coracoacromial tilt decreased significantly from Type I to Type III on both the right (F(2,17) = 25.81, p < 0.001, η² = 0.75) and left (F(2,17) = 33.61, p < 0.001, η² = 0.80) sides. Tukey analysis showed significant differences between Type I and both Type II and Type III, whereas Type II and Type III did not differ significantly after adjustment. Posterior acromial tilt showed even larger effect sizes, decreasing from 65.22 ± 1.41° in Type I to 52.40 ± 3.41° in Type III on the right (F(2,17) = 50.75, p < 0.001, η² = 0.86) and from 64.14 ± 2.44° to 50.80 ± 2.62° on the left (F(2,17) = 44.20, p < 0.001, η² = 0.84). These findings suggest that acromial orientation, in addition to categorical shape, substantially influences coracoacromial architecture [19]. Changes in tilt may alter the spatial relationship between the acromial undersurface and underlying rotator cuff structures during shoulder elevation and rotation [20]. Kocadal et al., Guo et al., Saha et al., and Balke et al. reported similar observations on the influence of acromial geometry on subacromial dimensions [21-24].

In contrast, DA did not differ significantly among acromial types on either the right (F(2,17) = 1.83, p = 0.191, η² = 0.18) or left (F(2,17) = 1.28, p = 0.305, η² = 0.13) side. Thus, despite pronounced differences in subacromial area and angular parameters, this isolated linear measurement did not discriminate among morphological groups. This finding suggests that the subacromial region may be better characterized by combined area and angular measurements than by a single linear dimension, consistent with the growing emphasis on multidimensional assessment of subacromial anatomy [25].

Acromial osteophytes were identified in 24 (60.0%) specimens, with the anteroinferior acromial region being a frequently observed location in the examined material. Previous osteological studies have similarly described acromial osteophytes as potentially relevant to subacromial morphology [26,27]. Osteophytes projecting from the inferior acromial surface could theoretically further reduce available clearance beneath the acromion. However, because complete numerical cross-tabulations according to osteophyte location, morphology, side, and acromial type were not available for formal inferential analysis, the present study does not establish a statistical association between osteophyte occurrence and a specific acromial morphology. These observations should therefore be interpreted descriptively.

Analysis of the 20 anatomically paired scapular sets demonstrated no significant right-left differences in coracoacromial tilt, DA, subacromial area, or coracoacromial distance. Posterior acromial tilt was the only parameter showing a significant side-wise difference, being greater on the right than on the left (67.56 ± 5.12° vs. 65.99 ± 5.43°; mean paired difference 1.57°, p = 0.0001). Although this may represent anatomical asymmetry or use-related remodeling, handedness and occupational history were unavailable, and a biological explanation cannot be established. Overall, the findings suggest that acromial morphological type is more strongly associated with subacromial architectural variation than laterality. Nevertheless, the relatively small subgroup sizes, particularly for Type III acromia, require interpreting the inferential findings as preliminary and confirming them in larger osteological, cadaveric, and imaging-based studies.

Limitations

This preliminary study included only 40 scapulae from a single museum collection, with Type III subgroups as small as four to six specimens, limiting statistical power and robust assessment of normality and variance assumptions. Donor age, sex, handedness, occupation, and clinical history were unavailable, precluding demographic and sex-stratified analyses. The subacromial area and angular measures were derived from two-dimensional photographs of a three-dimensional structure and may therefore be affected by positioning, projection, and parallax error. Soft tissues, dynamic scapular kinematics, rotator cuff pathology, and clinical impingement could not be assessed. Although one investigator repeated measurements three times, a formal intra-observer ICC was not prospectively calculated.

## Conclusions

The present study demonstrates a significant association between acromial morphology and subacromial architecture. Type III (hooked) acromion showed the smallest subacromial area, reduced oracoacromial tilt angle, reduced posterior acromial tilt, and a higher prevalence of osteophytes than Type I and Type II acromia. These morphometric alterations may contribute to subacromial narrowing and represent important anatomical risk factors for shoulder impingement. These findings provide valuable osteological evidence on how acromial morphology influences shoulder anatomy and may help clinicians, radiologists, and orthopedic surgeons understand structural predisposition to impingement-related shoulder disorders and plan appropriate interventions.

## References

[1] Alzahrani AH, Alqahtani B (2023). The association between shoulder pain and disability among Saudi office workers. Cureus.

[2] Linaker CH, Walker-Bone K (2015). Shoulder disorders and occupation. Best Pract Res Clin Rheumatol.

[3] Al Hammadi MI, Shah ZA, Rathod RK, Seddik MA (2025). Shoulder impingement pain syndrome: pathophysiology, diagnosis, and a review of current treatment strategies. Cureus.

[4] Umer M, Qadir I, Azam M (2012). Subacromial impingement syndrome. Orthop Rev (Pavia).

[5] Hanchard NC, Lenza M, Handoll HH, Takwoingi Y (2013). Physical tests for shoulder impingements and local lesions of bursa, tendon or labrum that may accompany impingement. Cochrane Database Syst Rev.

[6] McLean A, Taylor F (2019). Classifications in brief: Bigliani classification of acromial morphology. Clin Orthop Relat Res.

[7] Fallahpour N, Jamalipour Soufi G, Jamalipour Soufi K, Hekmatnia A (2023). Evaluation of the acromion variants in MRI and their association with rotator cuff injuries in non-traumatic patients. J Orthop.

[8] Altuntas Y, Ipek E, Balkanlı B, Alibakan G, Akbas H, Eren OT (2025). Association between acromial morphology and tear severity in arthroscopically confirmed rotator cuff tears. BMC Musculoskelet Disord.

[9] Birsel O, Çalışkan E, Eren İ, Yürük B, Özben H, Demirhan M (2024). The impact of acromial morphology on the localization and size of calcific tendonitis in the rotator cuff. Acta Orthop Traumatol Turc.

[10] Caffard T, Ludwig M, Kappe T, Reichel H, Sgroi M (2024). Acromial tilt, lateral acromial angle, and acromiohumeral interval as risk factors for full-thickness supraspinatus tendon ruptures. Cureus.

[11] Singroha R, Verma U, Malik P, Rathee SK (2017). Morphometric study of acromion process in scapula of north Indian population. Int J Res Med Sci.

[12] Pardeep PG, Kular PS, Ashwani AU, Parth PM, Kenisha KU (2025). Morphological variations of the acromion and their association with shoulder impingement and rotator cuff injury: a comprehensive clinical, radiological, and anatomical evaluation. J Contemp Clin Pract.

[13] Ravindranath Y, Abraham A, Thelekatt DJ, Oomen AT, Johnsia S (2018). Acromion - anatomic study of south Indian dry scapulae. Int J Contemporary Medical Res.

[14] Das SR, Dehury MK, Kumari BS (2021). An anatomical study of the acromian process of the scapula and its clinical implications. Int J Cur Res Rev.

[15] Aragão JA, Silva LP, Reis FP, Dos Santos Menezes CS (2014). Analysis on the acromial curvature and its relationships with the subacromial space and types of acromion. Rev Bras Ortop.

[16] Garving C, Jakob S, Bauer I, Nadjar R, Brunner UH (2017). Impingement syndrome of the shoulder. Dtsch Arztebl Int.

[17] Dhillon KS (2019). Subacromial impingement syndrome of the shoulder: a musculoskeletal disorder or a medical myth?. Malays Orthop J.

[18] Kaur R, Dahuja A, Garg S, Bansal K, Garg RS, Singh P (2019). Correlation of acromial morphology in association with rotator cuff tear: a retrospective study. Pol J Radiol.

[19] Li X, Xu W, Hu N, Liang X, Huang W, Jiang D, Chen H (2017). Relationship between acromial morphological variation and subacromial impingement: a three-dimensional analysis. PLoS One.

[20] Lawrence RL, Richardson LB, Bilodeau HL (2024). Effects of scapular angular deviations on potential for rotator cuff tendon mechanical compression. Orthop J Sports Med.

[21] Kocadal O, Tasdelen N, Yuksel K, Ozler T (2022). Volumetric evaluation of the subacromial space in shoulder impingement syndrome. Orthop Traumatol Surg Res.

[22] Guo X, Ou M, Yi G, Qin B, Wang G, Fu S, Zhang L (2018). Correction between the morphology of acromion and acromial angle in Chinese population: a study on 292 scapulas. Biomed Res Int.

[23] Saha S, Vasudeva N (2017). Morphometric evaluation of adult acromion process in North Indian population. J Clin Diagn Res.

[24] Balke M, Schmidt C, Dedy N, Banerjee M, Bouillon B, Liem D (2013). Correlation of acromial morphology with impingement syndrome and rotator cuff tears. Acta Orthop.

[25] Boulanger SM, Mahna A, Alenabi T, Gatti AA, Culig O, Hynes LM, Chopp-Hurley JN (2023). Investigating the reliability and validity of subacromial space measurements using ultrasound and MRI. J Orthop Surg Res.

[26] Chaimongkhol T, Benjachaya S, Mahakkanukrauh P (2020). Acromial morphology and morphometry associated with subacromial impingement syndrome. Anat Cell Biol.

[27] Hafezji HM (2021). Osteological study of morphometric analysis of acromion process and its implication in impingement syndrome. Nat J Clinical Anatomy.

